# Recent Advancements in Gene Therapy for Hereditary Retinal Dystrophies

**DOI:** 10.4274/tjo.41017

**Published:** 2017-12-25

**Authors:** Ayşe Öner

**Affiliations:** 1 Erciyes University Faculty of Medicine, Department of Ophthalmology, Kayseri, Turkey

**Keywords:** Gene therapy, hereditary retinal dystrophies, clinical studies

## Abstract

Hereditary retinal dystrophies (HRDs) are degenerative diseases of the retina which have marked clinical and genetic heterogeneity. Common presentations among these disorders include night or colour blindness, tunnel vision, and subsequent progression to complete blindness. The known causative disease genes have a variety of developmental and functional roles, with mutations in more than 120 genes shown to be responsible for the phenotypes. In addition, mutations within the same gene have been shown to cause different disease phenotypes, even amongst affected individuals within the same family, highlighting further levels of complexity. The known disease genes encode proteins involved in retinal cellular structures, phototransduction, the visual cycle, and photoreceptor structure or gene regulation. Significant advancements have been made in understanding the genetic pathogenesis of ocular diseases, and gene replacement and gene silencing have been proposed as potentially efficacious therapies. Because of its favorable anatomical and immunological characteristics, the eye has been at the forefront of translational gene therapy. Recent improvements have been made in the safety and specificity of vector-based ocular gene transfer methods. Dozens of promising proofs of concept have been obtained in animal models of HRDs and some of them have been relayed to the clinic. The results from the first clinical trials for a congenital form of blindness have generated great interest and have demonstrated the safety and efficacy of intraocular administrations of viral vectors in humans. This review summarizes the clinical development of retinal gene therapy.

## Gene Therapy in Hereditary Retinal Diseases

The concept of gene therapy emerged immediately after the discovery of DNA. The history of human gene therapy research goes back to the 1960s. However, initial attempts were unsuccessful due to a limited knowledge of gene expression and the inability to determine the best method for administering genetic material. Within the last 20 years, more than 1,500 clinical trials of gene therapy in various disease groups have been initiated.^1,2,3,4,5^

The eye is a common focus of gene therapy because it is a small, self-contained, and easily accessible organ with unique immunological properties. Furthermore, the noninvasive *in vivo* imaging techniques currently available enable fellow eyes to be used as controls for comparing treatment responses and outcomes, which provides a distinct advantage for gene therapy.^6,7^

Hereditary retinal dystrophies (HRD) are the disease group most studied in gene therapy research, though there is still no effective treatment. This rare disease group has an incidence of about 1/3,000. Retinitis pigmentosa (RP) is the most common HRD. The group also includes Leber congenital amaurosis (LCA), Stargardt macular dystrophy (SMD), Best’s macular dystrophy (BMD), and other even rarer retinal dystrophies. More than 200 genes are involved in the HRD disease group. There may be various mutations within the same gene, and these mutations may result in different phenotypes. This further complicates the genetic heterogeneity associated with these diseases. Recent advances have led to a better understanding of genetic pathogenesis and how gene therapies can be administered.^8,9,10^ Currently, gene therapy is implemented via vectors.

## Vectors Used in Gene Therapy

The vectors used in gene therapy are classified into two types: viral and nonviral vectors.

## Viral Vectors

**Retroviruses:** Retroviruses are a family of viruses with two single-stranded RNAs. The family includes seven genera: alpharetrovirus, betaretrovirus, gammaretrovirus, deltaretrovirus, epsilonretrovirus, lentivirus (LV), and spumavirus. Gammaretroviruses and more commonly LV have been used as retroviral vectors. The recombinant LV generated by eliminating many of the native viral genes has a large packaging capacity of 8 kilobases (kb), and can therefore carry multiple therapeutic proteins. It has also been shown that intraocular administration of LV does not stimulate an immune response. In addition, LVs can transduce and use the capsid glycoproteins of foreign viruses.^11,12^ Animal studies indicate that the subretinal use of LVs is safe in mice.^12,13,14,15^ However, these viruses have several disadvantages. LVs can lead to mutations in host cells. The production process of this vector is rather complicated and the end product is very large (~80-100 nm), which can affect its distribution in tissue.^12^

**Adenoviruses:** Adenoviruses are a family of virus with linear double-stranded DNA. There are 51 human serotypes of adenovirus, grouped from A to F. Viruses A2 and A5 in subgroup C are used in gene therapy and are nononcogenic. Adenoviruses do not affect the host cell cycle, and thus do not lead to cell death. Also, since the viral DNA does not integrate into the host cell’s genome, they do not cause mutagenesis in these cells. Adenovirus genes are stable in transduced cells, and they also have a large capacity. For these reasons, adenoviruses are a more attractive option for gene therapy. The virus is transformed into a vector by removing the sequences responsible for viral DNA replication. The packaging capacity can be increased based on the size of the removed section.^16,17,18^

**Adeno-associated viruses (AAV):** AAV is a parvovirus assisted by an adenovirus. Recombinant AAVs are currently the most frequently used virus in gene therapy. They show no pathogenicity and do not induce an inflammatory response. This is a major advantage in gene therapy. AAV contains a small DNA and its capacity is 4.7 kB. There are 9 serotypes of AAV (AAV1-AAV9). Of these, AAV2 is the most reliable and most efficiently transduces retinal pigment epithelium (RPE) cells.^19,20^ Preclinical and clinical studies have demonstrated the utility of AAV as a gene therapy vector for LCA.^21,22,23^

Viral vectors have higher gene transfer efficiency compared to other vectors. Although AAV2 shows efficient transduction of RPE cells, studies are ongoing to identify new vectors that will effectively target other retinal cells, particularly photoreceptors. Furthermore, the available capacity of AAV2 is inadequate for the treatment of larger genes. Nonviral vectors developed through studies on vector capacity have increased the number of target genes, and genes such as CEP290 in LCA, ABCA4 in SMD, and MYO7A in Type 1 Usher syndrome are currently within capacity. Nevertheless, larger genes such as USH2A still exceed the available capacity. Alternative therapies are being investigated in order to overcome this problem, and DNA nanoparticles have been proposed as a possible solution.^11,24^

## Nonviral Vectors

Nonviral factors were developed as the result of studies investigating vectors that were safer and had larger capacity than viruses. Among these, liposomes are the most studied. However, they were not found to be very effective in gene therapy culture studies.^25,26^

DNA nanoparticles developed as a result of studies on vector capacity are considered a potential solution to the capacity limitation. The capacity of DNA nanoparticles is 20 kb. This vector can carry the largest gene being studied, the USH gene, which is 15.6 kb in size and is responsible for Type 2A Usher syndrome. Studies have shown that these particles are safe when used in the lungs.^27^ Animal studies on their safety in the retina have also yielded favorable results. However, they are not as efficient for retinal cells as AAV, and therefore may require repeated subretinal injections.^28,29,30,31^

## Administration of Gene Therapy

The eye is an ideal organ for gene therapy. Firstly, it is small and enclosed. A small amount of vector is sufficient, which minimizes any toxic effects related to the vector. Moreover, the tight junctions between the RPE cells and the blood-retina barrier give rise to ocular immune privilege. The privileged intraocular microenvironment results in local inhibition of immune responses. These unique features prevent dissemination of the vector beyond the eye, thus avoiding the development of any systemic reactions to the vector. This greatly reduces the risk of systemic side effects. As retinal cells are post-mitotic, persistent gene expression can be achieved without interaction between genes. The numerous retinal dystrophy animal models have accelerated the process of evaluating treatment efficacy in preclinical trials. The structure of the eye facilitates treatment follow-up. The ability to observe the retina directly as well as with various *in vivo* imaging modalities allows noninvasive evaluation of gene therapy efficacy both in animal models and in humans.^32,33,34,35,36,37,38,39,40^ In addition, the bilateral and symmetric nature of dystrophies allows one eye to be used as a control to assess the effect of treatment on disease progression. Easy surgical access to the eye makes it possible for genetic material to be delivered directly to the desired ocular layer and target cell mass. Intraocular administration is usually performed via two routes: intravitreal and subretinal. In intravitreal injection, the therapeutic agent is dispersed in the vitreous, exposing the anterior retinal layers to the agent. In subretinal administration, the vector is injected between the RPE and the neurosensory retina, creating small, reversible pockets of detachment called blebs in the process. Diffusion of the agent is limited with intravitreal delivery. The vitreous inhibits diffusion first, followed by the internal limiting membrane and inner retinal layers. Therefore, subretinal administration is a more effective route because the targeted cell groups are located in the outer retina layers. After standard vitrectomy, the vector is administered in approximately 0.1 mm of fluid using a 39- or 41-gauge subretinal cannula. A site far from the large vessels, inside or outside the vascular arcade is selected as the injection site. The injections can also be done near the fovea. Treatment-related complications are mostly related to the surgery. No systemic complications related to the vector were reported in clinical trials. Ocular side effects associated with surgery can include subconjunctival hemorrhage, stinging, pain, and irritation. Although vector-related side effects are rare, hyperemia, photophobia, or decreased vision may occur.^12,30,41,42,43,44,45^

## Diseases in Which Gene Therapy Has Been Applied

**Leber Congenital Amaurosis:** LCA is a severe congenital retinal disease and is currently the most studied ophthalmologic disease in the field of gene therapy. Patients exhibit fundus signs, impaired light reflex, markedly reduced or absent response on electroretinography (ERG), and nystagmus. Vision loss starts at birth or within the first few years of life and results in total blindness during early adulthood. A number of gene mutations have been identified in LCA. To date, 20 different genes have been reported.26 One of these is RPE65 (a 65 kDa specific protein of retinal pigment epithelium), a gene that is expressed in RPE cells and encodes an isomerohydrolase which catalyzes the conversion of all-trans retinyl esters to 11-cis retinal. Without 11-cis retinal, opsins cannot capture light and convert it to electrical impulses. Loss of RPE65 disrupts the visual cycle, causing accumulation of retinyl esters in lipid droplets and an increase in lipofuscin granules in the RPE cells. The result is progressive retinal degeneration and loss of vision. Clinical trials of gene therapy for LCA have targeted RPE65 mutation.^31,32^

In mice with RPE65 gene mutation, AAV vector carrying this gene increases RPE transduction independently of disease stage. Injected RPE65 could be detected immunohistochemically even after 7 months. The treated mice showed normal retinal morphology and normal retinyl ester and rhodopsin (RHO) levels. Improvement of retinal functions was observed in ERG performed 2 months after treatment.^33^ Successful gene therapy outcomes depend on the presence of healthy photoreceptors.

Subretinal administration of AAV2 packaging the RPE65 gene provided retinal preservation and improved ERG responses in 1- to 2-month-old RPE65-knockout and rd12 mice.^34^

Gene therapy was also shown to provide visual improvements with a single injection in canine LCA2. Visual restoration began 2 weeks after injection, peaked at 3 months, and continued until 7 years.^35,36^ Studies conducted in various RPE65-mutant canine models have reported long-term improvements in vision and ERG findings.^37,38,39,40^ Although preclinical canine studies have shown safe outcomes, potential adverse effects such as dose-dependent retinal thinning may occur.^41^

RPE65-LCA clinical trials were initiated in 2007 following the encouraging results of animal studies. Clinical trials have demonstrated that AAV2-RPE65 gene replacement therapy is a surgically and immunologically safe treatment with no toxicity. Differences have been reported in terms of visual gains. This is related to the very severe vision loss caused by this disease.^42^ The longest outcomes reported in the literature to date includes data from 3 years of follow-up. The best results were achieved in young patients with better retinal responses. In a clinical trial of 5 patients followed for 3 years, visual and retinal functions were improved after a few months of treatment, and this improvement was maintained for 3 years. The patients showed reduced nystagmus frequency, improved multifocal ERG responses, and better fixation stability in microperimetry.^43^

Bainbridge et al.^44^ presented the 3-year outcomes of administering RPE65 packaged in rAAV2/2 in a phase I/II trial including 12 subjects. They reported increased retinal sensitivity but no significant difference in ERG findings. Three patients developed intraocular inflammation, while visual acuity deteriorated in 2 cases.^44^ In another phase I trial including 15 subjects, no systemic toxicity was observed and all patients showed varying degrees of improvement in visual acuity at the end of 3 years follow-up. Cone and rod sensitivity were increased in the treated eyes, while no differences were seen in the fellow eyes.^45^

A phase III study of AAV2/2-RPE65 gene therapy in patients about 3 years of age, supported by the Spark Therapeutics Biotechnology company, has been completed (NCT00999609). The study yielded successful outcomes, and in 2017 the same firm initiated procedures to obtain FDA approval for the drug, named voretigene neparvovec. The promising results in LCA clinical trials have led to gene therapy applications in other HRDs.

**Retinitis Pigmentosa:** Following the detection of genetic mutations in RP patients, studies related to gene therapy were initiated in these patients. There are two approaches to gene therapy in RP. The first approach is to package a normal copy of the affected gene into an AAV and administer by subretinal injection. The second approach is to inactivate the mutated gene.

RHO gene mutation was the first mutation detected in RP. The first approach to treatment aimed to accelerate proteosomal degradation in order to increase the function of the defective RHO gene. However, this method has not been successful in animal experiments conducted to date. Another alternative is treatment targeting RNA. The aim is to selectively destroy specific mRNA by using ribosomes to neutralize the mutant allele.^46,47,48,49,50^ Despite some success with this method in autosomal dominant (AD) RP, testing and therapeutically engineering all of the 120 different mutations identified in the RHO gene presents considerable economical and technical challenges.

Nagatsu et al.^51^ used rAAV to treat mutations in the cGMP phosphodiesterase (PDE) gene in patients with AD-RP. Intraocular administration of normal PDE in a mouse model preserved retinal functions and prevented photoreceptor degeneration. There have also been recent developments in gene therapy for X-linked RP. Mutation in the RPGTPase regulator gene (RPGR) has been detected in many X-linked RP patients. The RPGR gene is located on the X chromosome. Subretinal administration of RPGR packaged into AAV was shown to halt retinal degeneration in canine models.^52^

There are also studies of the RPE-expressed “human receptor tyrosine kinase MER” (MERTK) gene mutations seen in RP type 38. Subretinal administration of AAV2-MERTK in a mouse model prevented photoreceptor degeneration.^53,54^ Clinical trials in humans were initiated on the basis of consistent experimental results.

**Stargardt Macular Dystrophy:** SMD is a retinal degenerative condition caused by mutation in the ATP binding cassette subfamily member 4 (ABCA4; ABCR) gene. ABCA4 encodes the protein that allows the transmission of energy from photoreceptors. Mutation in this gene leads to photoreceptor degeneration and subsequent visual loss. Gene therapy trials in mice have yielded encouraging results. Naash^55^ reported that nanoparticle delivery of normal ABCA4 gene preserved vision in a mouse model of SMD.^56^

These results have also led to the initiation of human studies. Clinical trials targeting the ABCA4 gene mutation identified in SMD are ongoing (STGD1; NCT01367444).^57^

**Age-Related Macular Degeneration (AMD):** The complement regulatory protein CD59 reduces membrane attack complex formation, considered one of the causes of AMD, by 62%. CD59 delivered via gene therapy may prevent unregulated vascular growth.^58^

Soluble fms-like tyrosine kinase-1 (sFlt-1 or sVEGFR-1) is a tyrosine kinase protein and inactivates proteins that cause vascular growth. In 2011, a clinical trial investigating subretinal administration of rAAV.sFLT-1 in wet AMD was initiated. The study included 9 patients (6 in the study group and 3 controls) and no drug-related side effects were observed. Four patients in the study group responded well, and it was reported that this treatment is safe and has potential in the management of AMD.^59,60^

**Choroideremia:** Choroidemia is an X-linked progressive retinal degenerative disease. Affected male subjects have reduced night vision, peripheral vision loss, and total vision loss in the sixth decade. Mutation in the choroideremia (CHM) gene is associated with this disease. Encouraging results in preclinical trials led to a clinical trial of vectorized (AAV-REP1) normal CHM injected into the subretinal space. Two of the 6 subjects showed early improvement of visual acuity which was maintained for 3.5 years. There was no improvement in the fellow eyes. After 3.5 years, there was visual gain of up to 21 letters (4 lines) in the treated eyes, while the control eyes showed vision reduction of up to 18 letters.^61^

## CONCLUSION

Although the initial results of gene therapy seem promising, several questions remain to be answered. Viral vectors have higher gene transfer efficiency compared to other vectors. Although AAV2 shows good transduction efficiency for RPE cells, studies are ongoing to meet the need for vectors that effectively target other retinal cells, particularly photoreceptors. Furthermore, the endogenous 4.7 kb packaging capacity of AAV2 is inadequate for the treatment of larger genes. Nonviral vectors developed through studies on vector capacity have increased the number of target genes. As a result, expanded capacity vectors can now accommodate genes such as CEP290, ABCA4, and MYO7A, genes responsible for LCA, SMD, and Type 1 Usher syndrome, respectively. Nevertheless, larger genes such as USH2A still exceed the available capacity. Alternative therapeutic strategies such as DNA nanoparticles are currently being explored in order to circumvent this problem.
